# Survey of microsatellite clustering in eight fully sequenced species sheds light on the origin of compound microsatellites

**DOI:** 10.1186/1471-2164-9-612

**Published:** 2008-12-17

**Authors:** Robert Kofler, Christian Schlötterer, Evita Luschützky, Tamas Lelley

**Affiliations:** 1 University of Natural Resources and Applied Life Sciences, Department for Agrobiotechnology IFA-Tulln, Institute of Biotechnology in Plant Production, Konrad Lorenz Straße 20, 3430 Tulln, Austria; 2 Institut für Popluationsgenetik, Veterinärmedizinische Universitat Wien, Josef Baumann Gasse 1, 1210 Wien, Austria; 3 Umweltbundesamt, Spittelauer Lände 5, 1090 Wien, Austria

## Abstract

**Background:**

Compound microsatellites are a special variation of microsatellites in which two or more individual microsatellites are found directly adjacent to each other. Until now, such composite microsatellites have not been investigated in a comprehensive manner.

**Results:**

Our *in silico *survey of microsatellite clustering in genomes of *Homo sapiens*, *Maccaca mulatta*, *Mus musculus*, *Rattus norvegicus*, *Ornithorhynchus anatinus*, *Gallus gallus*, *Danio rerio *and *Drosophila melanogaster *revealed an unexpected high abundance of compound microsatellites. About 4 – 25% of all microsatellites could be categorized as compound microsatellites. Compound microsatellites are approximately 15 times more frequent than expected under the assumption of a random distribution of microsatellites. Interestingly, microsatellites do not only tend to cluster but the adjacent repeat types of compound microsatellites have very similar motifs: in most cases (>90%) these motifs differ only by a single mutation (base substitution or indel). We propose that the majority of the compound microsatellites originates by duplication of imperfections in a microsatellite tract. This process occurs mostly at the end of a microsatellite, leading to a new repeat type and a potential microsatellite repeat track.

**Conclusion:**

Our findings suggest a more dynamic picture of microsatellite evolution than previously believed. Imperfections within microsatellites might not only cause the "death" of microsatellites they might also result in their "birth".

## 1 Background

Microsatellites or simple sequence repeats (SSR) are DNA stretches consisting of a tandemly repeated short DNA motif (≤ 6 bp). Due to the special mutation mechanism of microsatellites termed "DNA replication slippage", these sequences often exhibit length hypervariability with respect to the number of motifs being repeated [reviews: [1-3]]. Owing to this hypervariability and an ubiquitous presence in genomes, microsatellites attracted much attention during the last decade and notably resulted in various genetic marker systems [4-6].

According to Chambers et al. [7] the following categories of microsatellites can be distinguished: Pure, Interrupted pure, Compound, Interrupted compound, Complex and Interrupted complex. In this survey we mainly refer to Compound and Interrupted compound microsatellites. This has to be distinguished from the term microsatellite cluster as used by Grover and Sharma [8] which refers to microsatellite rich regions. However, although microsatellites have first been described more than twenty years ago [9], their evolution is still not fully understood [2,3]. In particular imperfections within microsatellites have been the reason for much debate. Imperfections in the microsatellite tract are thought to interfere with replication slippage by limiting microsatellite size expansion [10-12]. If they accumulate in a microsatellite tract, they have even been proposed to cause the "death" of a microsatellite [13]. The complementary concept, the "birth" of a microsatellite was first introduced by Messier [14]. However, compound microsatellites, i.e. two or more microsatellites being found in close proximity, have been frequently reported in diverse taxa ranging from humans to plants [10,15-19]. Weber [10] estimated that, about 10% of the human microsatellites have a composite motif. Despite their abundance, compound microsatellites have not yet been studied in a comprehensive manner and very little is known about their origin and evolutionary dynamics.

This lack of knowledge about compound microsatellites is partly due to the difficulties involved by their identification using computer aided approaches. The analysis of compound microsatellites is additionally confounded by the fact that two microsatellites can be arranged in several different combinations [16,20]. For instance, the two microsatellites [AC]_*n *_and [AG]_*m *_can be found in four different arrangements. The [AG]_*m *_microsatellite might be located 5' or 3' to the [AC]_*n *_microsatellite and either the poly-TC or the poly-AG tract of the [AG]_*m *_microsatellite might be found on the same DNA strand as the poly-AC tract of the [AC]_*n *_microsatellite. For these reasons, four different motif standardizations were introduced by Kofler et al. [20] [see also Additional file 1].

Here we provide the first comprehensive survey of compound microsatellites in the fully sequenced genome of eight eukaryotic species. We surveyed the entire genomes as well as the coding sequence (cds) the 5' and the 3' untranslated region (5'-UTR and 3'-UTR) separately. We analyzed the genomes of five mammals (*Homo sapiens*, *Maccaca mulatta*, *Mus musculus*, *Rattus norvegicus*, *Ornithorhynchus anatinus*), a bird (*Gallus gallus*), a fish (*Danio rerio*) and a insect (*Drosophila melanogaster*). We show that 4 – 25% of all microsatellites are part of compound microsatellites and discuss the possible evolutionary mechanisms leading to the observed high frequency of compound micrsoatellites.

## 2 Results

### 2.1 Distance between microsatellites

**Figure 1:**
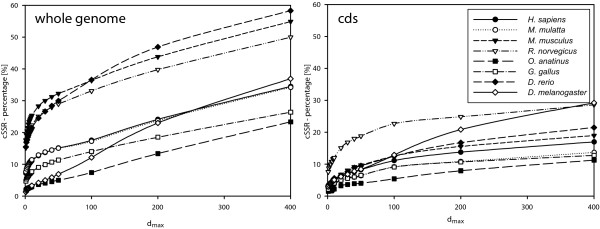
**Influence of *d*_*max *_to the cSSR-%**.

We define a compound microsatellite as an aggregation of at least two microsatellites with different motifs [partially standardized: see Additional file 1]. All identified microsatellites have a minimum length of 15 bp (see Material and Methods). Whether two or more adjacent microsatellites account as a compound microsatellite depends on the distance separating these microsatellites. In this work, microsatellites being separated by less than a maximum threshold *d*_*max *_were classified as compound microsatellite. For brevity, we termed individual microsatellites being part of such a compound microsatellite cSSR and the percentage of these microsatellites cSSR-%. We determined the impact of *d*_*max *_by measuring the proportion of microsatellite which could be classified as compound microsatellites (cSSR-%) with a given *d*_*max *_(Fig. 1). As expected, the number of compound microsatellites increases with *d*_*max*_, but the increase is not linear. While we observed species specific differences, the overall pattern is that around a *d*_*max *_of 50 bp an inflection point could be found, indicating a different behavior (Fig. 1). One difference between cds and whole genome is that for cds an upper boundary for the distance between two microsatellites exists, i.e. the total length of the cds.

### 2.2 Frequency of compound microsatellites

We quantified the compound microsatellite density in the different genomes by setting *d*_*max *_to 10 bp. Rodents and *D. rerio *had the highest proportion of microsatellite being classified as compound microsatellites (Table 1) whereas *D. melanogaster *and *O. latipes *had the lowest. Interestingly, for coding sequences no major differences were observed between the species (Table 1). Only *R. norvegicus *contained an exceptionally high cSSR-% in the cds (Table 1). In *D. melanogaster *this proportion was higher for coding sequences than for genomic sequences, indicating a more pronounced clustering in the cds than in non-coding sequences (Table 1). The impact of different SSR-search settings on the frequency of compound microsatellites can be found in Additional file 2 (Table S2).

**Table 1:** Frequency of compound microsatellites in the whole genome and in the coding sequence (cds).

	whole genome						coding sequence					
species	m.^1^	c.^2^	cSSR^3^	%^4^	m.d.^5^	c.d.^6^	m.^1^	c.^2^	cSSR^3^	%^4^	m.d.^5^	c.d.^6^
*H. sap*.	1 169 530	59 792	129 848	11.1	413.0	21.1	4 965	104	233	4.7	77.4	1.6
*M. mul*.	1 178 381	61 407	134 455	11.4	445.3	23.2	3 638	64	139	3.8	71.3	1.3
*M. mus*.	1 574 180	173 535	398 361	25.3	617.9	68.1	3 995	95	202	5.1	72.5	1.7
*R. nor*.	1 307 474	133 120	291 304	22.3	527.8	53.7	1 883	92	226	12.0	92.6	4.5
*O. anat*.	133 984	1 913	3 969	3.0	327.2	4.7	1 535	16	34	2.2	42.8	0.5
*G. gal*.	233 896	8 532	17 989	7.7	237.5	8.7	1 889	36	77	4.1	58.3	1.1
*D. rerio*	1 048 258	94 159	225 069	21.5	688.1	61.8	3215	86	180	5.6	72.0	1.9
*D. mel*.	44 600	714	1 457	3.3	376.9	6.0	4 168	105	213	5.1	145.6	3.7

^1^total number of microsatellites in DNA sequence space

^2^total number of compound microsatellites in DNA sequence space

^3^number of individual microsatellites being part of a compound microsatellite

^4^percentage of individual microsatellites being part of a compound microsatellite (cSSR-%)

^5^microsatellite density [m./Mbp]

^6^compound microsatellite density [c./Mbp]

*H. sap*.: *Homo sapiens*; *M. mul*.: *Macaca mulatta*; *M. mus*.: *Mus musculus*; *R. nor*.: *Rattus norvegicus*; *O. anat*.: *Ornithorhynchus anatinus*; *G. gal*.: *Gallus gallus*; *D. rerio*: *Danio rerio*; *D. mel*.:

### 2.3 Distribution of compound microsatellites within the genome of H. sapiens

The distribution of microsatellites is not homogeneous within genomes. For example, in *H. sapiens *and *M. musculus *an increase in microsatellite density toward the ends of the chromosomes was reported (in 2). We therefore investigated the distribution of compound microsatellites along the chromosomes. The SSR and the compound microsatellite densities were calculated with an overlapping sliding window approach using a window size of 5 Mbp and a step size of 1 Mbp.

**Figure 2:**
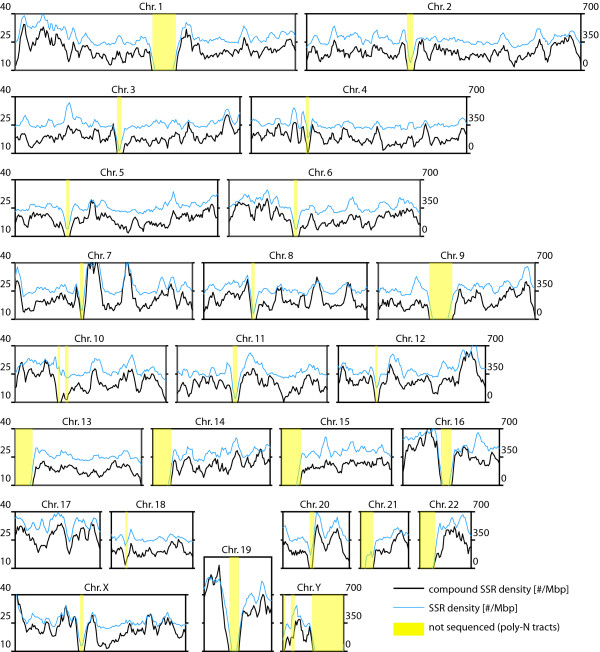
**Compound microsatellite density in the chromosomes of *H. sapiens *compared to the microsatellite density**. Regions which have not yet been sequenced are designated yellow. The scale of the compound microsatellite density is on the left hand side and the scale of the SSR density on the right hand side. The SSR and the compound microsatellite density were calculated with an sliding window approach using a window size of 5 Mbp and a step size of 1 Mbp.

Consistent with previous results, we show that the distribution of microsatellites varies along the chromosomes as well as between chromosomes of *H. sapiens *(Fig. 2). Generally, the distribution of compound microsatellites follows very closely the distribution of microsatellites. Nevertheless, some chromosome specific pattern could be detected. While for most chromosomes the peaks in compound microsatellite density follows the microsatellite density, on chromosome 15 only a relatively weak correspondence could be seen. Also on some chromosomes, the compound microsatellite pattern seems to be more pronounced than the microsatellite pattern (e.g. chromosome 8). Finally, the spacing between the lines indicating the microsatellite and compound microsatellite density differs among the chromosomes of *H. sapiens*, suggesting that the relative frequency of compound microsatellites differs among chromosomes (Fig. 2).

### 2.4 Parameters governing compound microsatellite density

Differences in compound microsatellite density can be caused by the parameters 'SSR density', 'species', 'chromosome' and 'recombination'. We tested which of these parameters has a significant influence on compound microsatellite density.

Due to the scarcity of species with sequenced Y-chromosomes only *H. sapiens*, *Pan troglodytes *and *M. musculus *were used for this analysis.

We observed that the parameters 'SSR-density' (CatReg: *p *< 0.001), 'species' (CatReg: *p *< 0.001) and 'chromosome' (CatReg: *p *< 0.001) have a highly significant influence on the compound microsatellite density. These three parameters are highly correlated with the compound microsatellite density (CatReg: *R*^2 ^= 0.94). Additionally, the relative contributions (rc) of these parameters to the regression could be identified. We found that 'species' (*rc *= 0.36) and 'chromosome' (*rc *= 0.38) have the strongest influence and that SSR density has a moderate influence (*rc *= 0.26). Because compound microsatellites are a subset of the total microsatellite repertoire, we modified our analysis and correlated the density of microsatellites that could not be classified as compound microsatellites with compound microsatellites. Again, 'species' (CatReg: *p *< 0.001),'chromosome' (CatReg: *p *< 0.001) and 'SSR density' (CatReg: *p *< 0.001) have a significant influence on compound microsatellite density and are highly correlated (CatReg: *R*^2 ^= 0.93) with the compound microsatellite density.

To determine the influence of recombination, we compared two groups of chromosomes (Y-chromosomes with chromosomes other than Y) with extreme differences in recombination rate and found no significant influence (CatReg: *p *= 0.214). To further test the influence of recombination we used the human recombination map published by Kong et al. [21] and compared the recombination frequencies with the compound microsatellite density and found only a very weak correlation (Linear regression: *R*^2 ^= 0.03) [see Additional file 3 and Additional file 4]

### 2.5 Compound microsatellite complexity

Compound microsatellites might contain different numbers of individual microsatellites (cSSRs). For example, the compound microsatellite [AC]_9 _[AG]_10 _contains two whereas the compound microsatellite [AC]_11 _[AG]_7 _[AC]_9 _three cSSRs. We call the former 'di-SSR' and the latter 'tri-SSR' compound microsatellite. Most compound microsatellites (≈ 87%) contain only two cSSRs (Table 2). The number of identified compound microsatellites decreases rapidly with an increasing complexity. However, very large compound microsatellites, containing more than eight cSSRs, can be found in many species (Table 2). We found the largest compound microsatellite in *D. rerio *chromosome 17, having 40 cSSRs. Only with a few exceptions the cds contains more than four cSSRs (Table 2). The complexity of compound microsatellites in the 5'-UTRs and 3'-UTRs is higher, but rarely exceeds three cSSRs [see Additional file 2: Table S7]. To test whether compound microsatellites originate from a nesting of microsatellites, i.e. secondary microsatellites emerging in the tract of primary microsatellites, we analyzed the percentage of tri-SSR compound microsatellites having the pattern: [m1]_*n*1 _[m2]_*n*2 _[m1]_*n*3 _where m1 and m2 are the motifs of the individual cSSRs [partially standardized: see Additional file 1]. In all eight species about 33% of the tri-SSR compound microsatellites exhibit this pattern [see Additional file 2: Table S11], which suggests that most (67%) tri-SSR compound microsatellites do not originate by a nesting of microsatellites.

**Table 2:** Compound microsatellite complexity in the whole genome and in the cds.

	whole genome								cds			
c.c.:^1^	2	3	4	5	6	7	8	≥ 9	2	3	4	≥ 5
*H. sap*.	51 997	6 096	1 198	335	106	41	7	12	81	21	2	0
*M. mul*.	52 796	6 565	1 389	433	155	49	10	10	53	11	0	0
*M. mus*.	137 237	26 551	6 561	2 080	652	241	99	114	84	10	1	0
*R. nor*.	113 077	16 505	2 632	607	170	78	19	32	72	11	5	4
*O. anat*.	1 791	105	13	4	0	0	0	0	14	2	0	0
*G. gal*.	7 782	610	115	17	6	2	0	0	32	3	1	0
*D. rerio*	71 280	15 703	4 163	1 641	592	336	143	301	78	8	0	0
*D. mel*.	685	29	0	0	0	0	0	0	102	3	0	0

^1^compound microsatellite complexity

Complexity refers to the number of individual microsatellites constituting the compound microsatellite. All values are in counts

### 2.6 Aggregation of microsatellites

To test whether the occurrence of compound microsatellites can be attributed to mere chance, we determined whether microsatellites tend to aggregate with respect to an assumed random distribution of microsatellites in the genome.

For simplicity we confine this analysis to pairs of adjacent microsatellites and introduce the technical concept of SSR-couples. SSR-couples are each two adjacent microsatellites being separated by less than 10 bp (*d*_*max*_), which can be part of a more complex compound microsatellite. For example a tri-SSR compound microsatellite could be viewed as two overlapping SSR-couples. SSR-couples containing two microsatellites with an identical motif were not considered [partially standardized: see Additional file 1].

Table 3 shows that SSR-couples are significantly overrepresented in the whole genome (Poisson Distribution: *p *≈ 0) as well as in the cds (Poisson Distribution: *p *< 10^-22^) of the eight species. Although less abundant than in the entire genome, SSR-couples are significantly overrepresented in the 5'-UTR and 3'-UTR [see Additional file 2: Table S8]. Since we observed regional variation in microsatellite and compound microsatellite densities in all chromosomes (Fig. 2) [see Additional file 5] we conducted this analysis in all eight species separately for each sliding window (size 5 Mbp). We found that the number of observed SSR-couples significantly deviates from the expected number in each sliding window (Poisson Distribution: *P *< 10^-4^) [see Additional file 5]. Therefore, our results do not support the hypothesis of a random distribution of microsatellites. Interestingly, the overrepresentation of SSR-couples in the cds is consistently more than twofold higher than in the whole genome (Table 3) whereas it is the lowest in the 5'-UTR and 3'-UTR [see Additional file 2: Table S8].

**Table 3:** Overrepresentation of SSR-couples in the whole genome and in the cds.

	whole genome				cds			
	obs.^1^	exp.^2^	or.^3^	P^4^	obs.^1^	exp.^2^	or.^3^	P^4^
*H. sap*.	69 670	4 488	15	0^5^	129	4	36	0^5^
*M. mul*.	72 780	4 800	15	0^5^	74	2	30	3E-82
*M. mus*.	223 973	9 526	23	0^5^	107	3	40	0^5^
*R. nor*.	157 300	6 639	23	0^5^	134	2	81	0^5^
*O. anat*.	2 052	399	5	0^5^	18	1	28	6E-22
*G. gal*.	9 435	512	18	0^5^	41	1	40	9E-52
*D. rerio*	130 012	7 026	18	0^5^	93	2	42	0^5^
*D. mel*.	743	164	4	0^5^	108	4	24	0^5^

^1^observed number of SSR-couples

^2^expected number of SSR-couples with respect to a random distribution of microsatellites within DNA sequence space

^3^overrepresentation (obs./exp.)

^4^significance of the overrepresentation based on a Poisson Distribution

^5^*p *< 1*E *- 99

### 2.7 Motifs of compound microsatellites

To answer whether there is any motif preference in the composition of compound microsatellites, we examined which microsatellites are most frequently found in close proximity, e.g. whether the microsatellite [AC]_*n *_is more frequently associated with the microsatellite [AG]_*n *_than with any other microsatellite motif. For simplicity, we confined this analysis again to SSR-couples. We define SSR-couples having the form [m1]_*n *_[m2]_*n *_as SSR-couples of motif m1–m2, e.g.: the SSR-couple [AT]_12 _[AC]_9 _has the motif AT-AC [fully standardized: see Additional file 1].

Additionally we examined the conformation of the SSR-couples. Each microsatellite consists of two tracts, for example a [AC]_*n *_microsatellite consists of a poly-AC and a poly-TG tract on the complementary strand. The SSR-couple [AC]_8 _[AG]_9 _can be found in two conformations, the poly-AC tract of the [AC]_8 _microsatellite may either be found on the same or on the complementary DNA-strand as the poly-AG tract of the [AG]_9 _microsatellite. We call the former plus-conformation and the latter minus-conformation [see Additional file 1]. Table 4 shows the characteristics of the most abundant SSR-couple motifs in the whole genome of the eight species and Table 5 shows equivalent information for the cds. [see Additional file 2: Table S9 in the 5'-UTR, Table S10 in the 3'-UTR]. These tables also contain the conformation and the proposed genesis of each SSR-couple.

**Table 4:** Characteristics and probable genesis of the most abundant SSR-couples in the whole genome

	*H. sapiens*					*M. mulatta*			
motif	obs.^1^	or.^2^	%plus^3^	gen.^4^	motif	obs.^1^	or.^2^	%plus^3^	gen.^4^
AT-AC	5 975	134	(100)	s	AAAG-AAGG	5 659	870	100	s
AC-AG	5 456	173	28	s	AC-AG	5 628	169	31	s
AAAG-AAGG	5 149	844	100	s	AT-AC	5 205	173	(100)	s
A-AAAG	4 401	37	100	s	A-AAAG	4 481	32	100	s
AAGG-AGGG	4 325	2265	100	s	AAGG-AGGG	4 456	2311	100	s
A-AT	4 234	25	(100)	s	A-AT	3 505	26	(100)	s
A-AAAAG	3 263	50	100	s	A-AAAAG	3 296	42	100	s
AT-AG	2 025	133	(100)	s	AG-AAAG	2 582	222	100	s
AG-AAAG	1 750	161	100	s	AT-AG	1 618	146	(100)	s
AAAT-AAAAT	1 106	58	99	s	A-AG	1 547	11	95	s

	*M. musculus*					*R. norvegicus*			
AC-AG	38 006	94	48	s	AC-AG	42 254	103	50	s
AAAG-AAGG	15 941	943	100	s	AT-AC	7 963	48	(100)	s
AT-AC	11 459	69	(100)	s	AAAG-AAGG	6 248	1000	100	s
AAG-AGG	9 439	1983	100	s	AAG-AGG	4 662	1962	100	s
AAGG-AGGG	8 829	913	100	s	AC-ACAG	4 107	50	95	s
AG-AAAG	8 350	129	100	s	AG-AGGG	3 993	184	100	s
AG-AGGG	7 645	206	100	s	AG-ACAG	3 372	110	99	s
AAAC-AAAAC	3 877	59	100	s	AC-CG	3 013	308	(100)	s
AG-AAGG	3 763	83	100	?	AT-AG	2 654	43	(100)	s
A-AAAT	3 623	37	98	s	AC-ACGC	2 554	168	99	s

	*O. anatinus*					*G. gallus*			
AC-AG	476	267	4	s	A-AAAG	530	48	99	s
AT-AC	175	111	(100)	s	AAAC-AAAAC	412	74	100	s
AAT-ATC	113	11	14	s	AAAG-AAGG	341	1209	100	s
AT-AG	79	87	(100)	s	AT-AC	309	173	(100)	s
AAT-AATG	76	1	37	c	A-AC	293	21	98	s
AAT-AAT	71	1	(0)	s	AAC-AAAC	266	72	99	s
AATG-ACTG	65	38	98	s	A-AAAC	260	6	95	s
AATG-ATCC	37	79	0	s	AAGG-AGGG	254	5492	100	s
AATC-AATG	31	3	26	c/s	A-AAAAG	228	45	99	s
AG-AAAG	31	301	100	s	A-AAG	223	95	100	s

	*D. rerio*					*D. melanogaster*			
AT-AC	21 990	63	(100)	s	AAC-AGC	45	53	100	s
A-AT	11 172	48	(100)	s	A-AAT	23	20	57	s
ATAG-ACAG	10 370	1516	100	s	AT-AC	18	5	(100)	s
ATAG-ATCC	6 503	497	0	s	AT-ATAC	17	25	(100)	s
AAT-AAT	5 910	38	(0)	r/s	ATC-AGC	15	29	93	s
AT-ATAC	4 587	230	(100)	s	ACC-AGC	12	42	100	s
AC-AG	3 830	49	26	s	AAT-AAAT	12	68	100	s
AAT-ACT	3 685	316	84	s	AGC-AGG	8	29	88	s
AAT-AAC	3 624	204	91	s	AGC-AACAGC	7	69	100	s
AT-AAAT	2 973	17	(100)	s	AT-AAT	7	11	(100)	s

^1^observed number of SSR-couples having the given motif

^2^overrepresentation

^3^percent of the SSR-couples found in the plus-conformation (see Text). Values in brackets indicate that only the specified conformation is feasible (e.g.: SSR-Couples containing self complementary microsatellites)

^4^suggested genesis of the SSR-couple: c: chance; r: recombination; s: slippage; ?: unknown

**Table 5:** Characteristics and probable genesis of the most abundant SSR-couples in the cds

	*H. sapiens*					*M. mulatta*			
motif	obs.^1^	or.^2^	%plus^3^	gen.^4^	motif	obs.^1^	or.^2^	%plus^3^	gen.^4^
AGC-CCG	20	74	20	s	AAC-AGC	12	2 244	100	s
AAC-AGC	18	1 913	100	s	AGC-CCG	8	61	25	s
AAG-AGG	10	133	100	s	AAG-AGG	7	160	100	s
AGG-CCG	9	38	22	s	AAAG-AAGG	5	> 10^4^	100	s
AAG-ATC	6	428	0	s	ACC-CCG	4	134	100	-
ACC-CCG	5	73	80	s	AGC-AGCTCC	3	367	100	-
AGCCTG-AGGCCC	4	> 10^4^	0	-	AGG-AAGAGG	3	508	100	-
AGC-AGCCTG	4	2 381	0	-	A-AAG	3	122	100	-
AGC-AGG	4	12	100	-	AGC-AGG	3	15	100	-
ACG-AGG	3	419	100	-	AGG-CCG	2	19	0	-

	*M. musculus*					*R. norvegicus*			
AAG-AGG	13	210	100	s	AACC-ATCC	16	> 10^4^	100	s
AAC-AGC	10	751	100	s	AT-AC	12	2 473	(100)	s
AC-AG	7	5 655	43	s	AAG-AGG	12	353	100	s
CCG-AGCCGG	6	2 937	100	s/?	AAAG-AAGG	9	> 10^4^	100	s
AGC-AGGCCC	6	732	100	?	AG-AAAG	9	3520	100	s
ACC-CCG	5	121	100	s	AC-AG	7	481	86	s
AAAG-AAGG	5	> 10^4^	100	s	CCG-AGCCGG	5	4 828	100	s/?
AGC-CCG	4	25	0	-	AGG-CCG	4	86	0	-
AGG-CCG	3	23	67	-	AG-AAGG	4	2 347	100	-
AAG-AAAAG	2	1 159	100	-	AG-ACAG	4	9 387	100	-

	*O. anatinus*					*G. gallus*			
AAC-AGC	2	4 265	100	-	AAAG-AAGG	5	> 10^4^	100	s
AGC-AATG	2	262	100	-	ACG-AGC	4	1 260	100	-
ACG-AGG	2	319	100	-	A-AAAG	4	2 605	100	-
ACT-AGG	2	3 828	0	-	ACC-AGG	3	121	0	-
AC-AG	1	1 866	0	-	AAG-AGG	3	107	100	-
AATG-AAGG	1	3 445	100	-	AAGG-AGGG	2	> 10^4^	100	-
AGC-ACACC	1	2 843	100	-	CCG-CCGCG	2	2 085	100	-
AG-AAAG	1	7 464	100	-	AGC-CCG	1	21	0	-
AAC-ACACC	1	> 10^4^	100	-	ACCGC-AGCGG	1	> 10^4^	0	-
ATC-ACG	1	1 464	0	-	AGC-AGG	1	12	100	-

	*D. rerio*					*D. melanogaster*			
AAC-AGC	12	788	100	s	AAC-AGC	36	62	100	s
AAT-AAAT	9	4 273	100	s	AGC-CCG	8	40	75	s
AACC-ATCC	6	> 10^4^	100	s	ACC-AGC	7	19	100	s
AC-AC	6	41	(0)	r/?	AGC-AGG	5	13	80	s
ATCC-ACGG	6	> 10^4^	0	s	AAT-AAC	4	315	100	-
ATC-ACG	4	5 622	0	-	AAC-ATC	4	140	100	-
AAG-ATC	4	113	0	-	ATC-AGC	4	24	100	-
ATC-AGG	4	58	0	-	ACG-AGG	3	240	100	-
AAT-ACT	3	9 081	100	-	AGC-AACAGC	3	31	100	-
ACC-AGC	3	126	0	-	AAC-ACC	3	47	100	-

^1^observed number of SSR-couples having the given motif

^2^overrepresentation

^3^percent of the SSR-couples found in the plus-conformation (see Text). Values in brackets indicate that only the specified conformation is feasible (e.g.: SSR-Couples containing self complementary microsatellites)

^4^suggested genesis of the SSR-couple: c: chance; r: recombination; s: slippage; ?: unknown

In the whole genome of all eight species the most abundant SSR-couple motifs are AT-AC, AC-AG and AAAG-AAGG (Table 4). Different SSR-couple motifs are overrepresentated to different degrees (Table 4). The SSR-couple motif AAGG-AGGG, for instance, is 1000-times more abundant than expected by chance.

In contrast, SSR-couples containing an [A]_*n *_microsatellite usually are only about 40 fold overrepresented. A few SSR-couples have an overrepresentation of ≈ 1, which suggests that they have emerged by chance. Most SSR-couples, however, are mainly found in only one of the two possible conformations (Table 4), i.e they are conformation specific. For example, SSR-couples with the motif AG-AAAG are always in the plus conformation (Table 4). Conformation specificity of SSR-couple motifs suggests that these SSR-couples have not arisen by chance. Only SSR-couples having the motif AC-AG are frequently found in both conformations (Table 4).

SSR-couples containing two microsatellites with complementary motifs such as [CTG]_13_- [CAG]_67 _have been proposed to arise from recombination between homologous microsatellites [22]. Only [AAT]_*n*_-[ATT]_*n *_(motif: AAT-AAT) SSR-couples in *D. rerio *and *O. anatinus *have such complementary motifs (Table 4). Instead, most SSR-couples contain two microsatellites with very similar motifs (Table 4) differing by a single mutation (base substitution or indel) in more than 90% of cases. Hence, only a single mutation would be required for a transformation of one motif into the other. While this is obvious for SSR-couples with motifs like AAGG-AGGG, SSR-couples with motifs like AG-AAAG might require further explanation. The SSR-couple AG-AAAG could in fact also be depicted as AGAG-AAAG, which illustrates how only one base substitution is required to transform the repeat motif AG into the motif AAAG. In another example, SSR-couples with the motif ATAG-ATCC in *D. rerio *are only found in the minus conformation. The two individual microsatellite motifs of the plus conformation, ATAG and ATCC, differ by two base substitutions, whereas the two motifs of the minus conformation, ATAG and ATGG, only differ by a single base substitution. These ATAG-ATCC SSR-couples are only found in the conformation which requires the fewest base substitution to transform one motif into the other, i.e the minus conformation. In particular SSR-couples with the motif AC-AG provide interesting insight into the origin of compound microsatellites. Since individual microsatellite motifs of the plus and the minus conformation only differ by a single base substitution (plus: AC ⇌ AG; minus: AC ⇌ TC). Interestingly, both conformations can be found in all examined species with relativly equal frequencies (balanced conformation, Table 4). Overall, we found that almost all SSR-couples contain two cSSRs with highly similar motifs. These motifs will typically require only a single base substitution for transformation into the other motif. This suggests that most of the cSSRs forming a compound microsatellite are derived from a preexisting microsatellite.

## 3 Discussion

We present the first comprehensive survey of compound microsatellites in eight fully sequenced eukaryote genomes. The most influential parameter on the number of identified compound microsatellites is the maximum distance between two adjacent microsatellites. If microsatellites were randomly distributed, a linear increase of cSSR frequency with *d*_*max *_would be expected. Nevertheless, we observed that it is more likely to have two microsatellites in close proximity. We note, however, that defining the optimal *d*_*max *_is somewhat complicated for microsatellites carrying imperfections. Due to partially incomplete SSR-search, not always identifying the whole microsatellite tract, neighboring microsatellites might not be recognized as a compound microsatellite. Therefore, the choice of *d*_*max *_should aim to allow a certain degree of inaccuracy in the SSR-search and at the same time provide the maximum sensitivity for the identification of compound micrsosatellites. We account for this uncertainty by allowing for mismatches in the SSR-search and by using a *d*_*max *_of 10 bp.

### 3.1 Microsatellite clusters: frequency and general features

To our knowledge, the only estimate of compound microsatellites frequency was published by Weber [10] who estimated that about 10% of all *H. sapiens *microsatellites have a compound motif. Given the limited amount of sequence information available at that time, this estimate corresponds remarkably well with our results based on the complete genome.

In *H. sapiens*, about 11% of all microsatellites are part of a compound microsatellite (Table 1). The large majority of these compound microsatellites is located in intergenic regions. The distribution of compound microsatellites in *H. sapiens *is fairly homogeneous throughout all chromosomes, i.e. no clustering at the telomeres and around the centromeres could be observed (Fig. 2). Compound microsatellites are 4 – 23 fold overrepresented in the whole genomes of eight fully sequenced species (Table 3), which is highly significant (Poisson Distribution: *P *< 0.001). Bachtrog et al. [23] reported similar results in an analysis of 13 Mbp of the *D. melanogaster *genome that microsatellites tend to aggregate and significantly deviate from a random distribution within the investigated sequence.

Interestingly, despite their rare occurrence, compound microsatellites are most overrepresented in the cds (Table 3) which may indicate that these compound microsatellites are conserved because of an involvement in cellular processes. A recent review by Kashi and King [24] for example suggested that compound microsatellites might be involved in the regulation of *avpr1a *which influences social behaviour in voles. In the cds however, most SSR-couples contain microsatellites having motifs of length three or six base pairs (Table 5). This is not surprising, as these microsatellites do not cause a shift in the reading frame in case of a slippage event [25].

Three main parameters governing compound microsatellite density can be identified: 'species', 'chromosome' and the overall 'SSR-density'. These three parameters are highly correlated with compound microsatellite density (*R*^2 ^= 0.94). The parameters with the most significant influence are 'chromosome and 'species', accounting for 38% and 35% of the observed variation in compound microsatellite density, respectively. We hypothesize that the rate of base substitutions and the efficiency of the mismatch repair system are responsible for the high influence of the species, since these processes has been identified as to be crucial for the evolution and stability of microsatellites in general [1-3].

The significant differences in compound microsatellite density between chromosomes (CatReg: *p *< 0.001) were not expected, we could only speculate about the processes which might be responsible for this differences.

### 3.2 Genesis of compound microsatellites: Recombination

Jakupciak and Wells [22] showed that 'illegitimate' recombination involving an inversion between two homologous microsatellites may create compound microsatellites consisting of two microsatellites with self complementary motifs such as [CTG]_13 _[CAG]_67_. Assuming that compound microsatellites predominately originate through the process described by Jakupciak and Wells [22] and further assuming that 'illegitime' recombination rates are positively correlated with normal recombination rates, the Y chromosomes ought to have significantly less compound microsatellites than the autosomes. This was not confirmed by our results, which suggest that recombination does not have a significant influence on compound microsatellite density (CatReg: *p *= 0.214 and Linear Correlation: *R*^2 ^= 0.03). Moreover SSR-couples created by recombination will exhibit a distinctive pattern: they (i) should be overrepresented compared to a random distribution of microsatellites in the genomes, (ii) they should only be found in the minus-conformation, (iii) the motifs of the two microsatellites forming a SSR-couple should have identical length (e.g.: AC-AG), (iv) and these two motifs should be mutually complementary (summary in Table 6; abbr.: 'r'). Table 4 demonstrates that only very few SSR-couples show this pattern, therefore we suggest that SSR-couples formed by 'illegitimate' recombination are rare and most SSR-couples (and thus compound microsatellites) are created by processes other than recombination.

**Table 6:** Overview of the recognition pattern of different mechanism potentially generating SSR-couples

proposed origin	overrepresentation	conformation	motif length	motif similarity
chance (c)	none (low)	balanced	none required	none required
recombination (r)	medium	unbalanced – minus	equal	reverse complement
slippage (s)	high	unbalanced	equal (stepwise equal)	high

### 3.3 Genesis of compound microsatellites: Random events

The highly significant overrepresentation of SSR-couples (Table 3) indicates that only a minor fraction of the compound microsatellites can be attributed to a coincidental emergence of a microsatellite in the proximity of an already existing one. SSR-couples formed by chance should also show a distinctive pattern: (i) they should not be overrepresented, (ii) they should have a balanced conformation (e.g. 50% plus and 50% minus conformation) and (iii, iv) the motifs of the individual microsatellite forming these SSR-couples need not to be similar in length and sequence (summary in Table 6; abbr.: 'c'). A high overrepresentation and an unbalanced conformation are strong indications that the respective SSR-couples are not a product of chance. Table 4 shows that only the SSR-couples having the motif AAT-AATG in *O. anatinus *exhibit both a low overrepresentation and a relatively balanced conformation. Therefore our results suggest that the majority of the SSR-couples can not be attributed to a coincidental emergence of a microsatellite in the proximity of an already existing one

### 3.4 Genesis of compound microsatellites: Imperfections within microsatellites

We found that the graphs of the microsatellite and compound microsatellite density have a highly similar overall shape (Fig. 2) and that the SSR-density is significantly correlated with the compound microsatellite density (CatReg: *p *< 0.001). Three scenarios for this high interdependence between microsatellite and compound microsatellite density are in theory possible. First, recombination between homologous microsatellites might lead to elevated compound microsatellite densities in genomic regions having a high SSR density. Second, an increased SSR density might increase the frequency of adjacent SSRs due to chance. Third, imperfections in the tract of microsatellites may be the origin of compound microsatellites [26-29]. Since we already excluded the first two scenarios only the hypothesis that imperfections within microsatellites may give rise to compound microsatellites remains as the most probable explanation. Possible molecular mechanism explaining how imperfections within microsatellites may generate compound microsatellites have already been discussed [27,28]. Basically, mutations within a microsatellites generate an imperfect motif repeat which may be duplicated tandemly due to replication slippage [27-29], thus generating a 'proto' compound microsatellites. This 'proto' compound microsatellites consist of a long and a short microsatellite which may have as few as two adjacent repeat units. Two motif repeats are already sufficient for independent expansion of the microsatellite by replication slippage or indel-like events [30,31]. After adequate expansion of the short microsatellite, the primary combined with the secondary microsatellites will be regarded as compound microsatellite. However, replication slippage events involving the imperfect motif repeat may also span several motif repeats in which case the motif of the primary and the secondary microsatellite will have a stepwise length difference (e.g.: AC-AGAC, AC-AGACAC, A-AAAG). The SSR-couples generated by the duplication of imperfect motif repeats should have a distinctive pattern: (i) they should be highly overrepresented since a single mutation, followed by a slippage event is sufficient for the formation of the proto compound microsatellite; (ii) these SSR-couples should mostly be found in one conformation, either plus or minus; (iii) the motif length of the primary and the secondary microsatellite should either be equal or differ in a stepwise manner; and (iv) the motifs of the primary and the secondary microsatellite should be similar, mostly differing only by a single mutation (iv) (summary in Table 6; abbr.: 's'). The majority of the SSR-couples exhibits this pattern (Table 4). Therefore we suggest that DNA replication slippage is the predominant mechanism generating compound microsatellites. Compared to other mammals, *M. musculus *(25%) and *R. norvegicus *(23%) have a very high number of cSSRs. Huttley et al. [32] showed that rodents have a 14% higher substitution rate than primates, which may cause elevated numbers of imperfections in primary microsatellites. Replication slippage involving these imperfections might thus be responsible for the high frequency of cSSRs in rodents.

### 3.5 Refining the theory of the origin of compound microsatellites

In the previous section we proposed that imperfections within microsatellite tracts serve as seeds for most compound microsatellites. It might further be asked whether secondary microsatellites preferentially emerge at certain position within the tract of primary microsatellites.

We observed that the majority of compound microsatellites consist of two cSSRs. If a secondary microsatellite would emerge in the middle of a primary microsatellite, a tri-SSR compound microsatellite would result. For instance, if an [AT]_*n *_microsatellite would originate within an [CA]_*n *_microsatellite a compound microsatellite having the form [CA]_*n *_[AT]_*n *_[CA]_*n *_would result. This example illustrates that, first the resulting compound microsatellite would be a tri-SSR compound microsatellite and that second the two microsatellites flanking the central microsatellite would share the same motif. Only about 13% of the compound microsatellites contain three or more microsatellites (Table 2). Therefore we suggest that most secondary microsatellites emerge at the ends of primary microsatellites.

To further test this hypothesis we investigated the number of tri-SSR compound microsatellites having the pattern [m1]_*n *_[m2]_*n *_[m1]_*n *_(partially standardized [see Additional file 1]), i.e. having a secondary microsatellite nested within a primary microsatellite and found that only about 33% of the tri-SSR compound microsatellites have this pattern [see Additional file 2: Table S11].

This suggests that most tri-SSR compound microsatellites originate by two independent 'births' of secondary microsatellites, rather than a nesting of microsatellites. What mechanism could be responsible for this observed bias? How is it possible that secondary microsatellites preferentially emerge at the ends of primary microsatellites? Brohede and Ellegren [17] found that the substitution rate within microsatellites is lowest in the center and highest at the ends of the microsatellite tracts. Since, imperfections within microsatellites are the source of secondary microsatellites, the mutational bias described by Brohede and Ellegren [17] might result in a biased origin of a secondary microsatellite at the ends of primary microsatellites.

### 3.6 Conclusion

In this work we present the frequency, general features and distribution of compound microsatellites in the fully sequenced genomes of eight eukaryotes. We show that as much as 4–25% of all microsatellites may be part of compound microsatellites. We propose that the majority of compound microsatellites is generated by tandem duplications of imperfect repeats, mainly at the end of primary microsatellites.

This work reveals a new aspect in microsatellite evolution thus extending the present views on microsatellite evolution that suggests that imperfections restrict microsatellite size expansion [11] or even lead to their 'death' [13]. Indeed, without contradicting these observations our results suggest that imperfection within microsatellites may as well be the 'birth' of new microsatellites. With up to 25% of microsatellites part of a compound microsatellite, it becomes clear that this phenomenon may be another driving force of microsatellite evolution and thus should not be neglected in future studies.

## 4 Methods

### 4.1 Sequence

The genomic pseudomolecules of *Homo sapiens *(assembly: NCBI36; release: 42), *Pan troglodytes *(assembly:CHIMP2.1; release 42), *Maccaca mulatta *(assembly: MMUL 1; release: 45), *Mus musculus *(assembly: NCBIM36; release: 42), *Rattus norvegicus *(assembly: RGSC3; release: 45), *Ornithorhynchus anatinus *(assembly: OANA5; release: 48), *Gallus gallus*(assembly: WASHUC2; release: 42), *Danio rerio *(assembly: ZFISH6; release: 42) and *Drosophila melanogaster *(assembly: BDGP4.3; release: 42) were downloaded from the Ensembl ftp-server .

Since sequence information of the Y-chromosome is not available for all examined species, only the autosomes and the X-chromosomes were used unless stated in the text. Non-chromosomal DNA was not considered. The 5' untranslated region (5'-UTR), coding sequence (CDS) and 3' untranslated region (3'-UTR) were obtained with Ensembl BioMart . The sequences obtained with BioMart, were pretreated to remove empty sequences and to ensure that each sequence has an unique identifier (fasta ID). This is an important prerequisite for the identification of compound microsatellites with SciRoKo. Table 7 shows detailed information for the examined sequences.

**Table 7:** Features of the DNA sequences used in this work.

		*H. sap*.	*M. mul*.	*M. mus*.	*R. nor*.	*O. anat*.	*G. gal*.	*D. rerio*	*D. mel*
whole genome	#^1^	23	21	20	21	19	31	25	6
	nt^2^	2 832	2 646	2 547	2 477	409	984	1 523	118

cds	#^1^	41 997	35 463	36 240	14 026	26 818	22 013	31 623	17 242
	nt^2^	64	51	55	20	36	32	44	28

5'-UTR	#^1^	31 051	16 925	27 882	11 269	1 737	10 353	8 717	14 466
	nt^2^	9	4	7	3	0.2	1	1	4

3'-UTR	#^1^	28 839	17 284	27 124	11 441	2 436	12 444	8 615	11 351
	nt^2^	31	12	28	7	1	6	5	5

^1^number of individual fasta sequences

^2^length of the sequence, not considering the character 'N')

All data were obtained with the tool 'Seq-CC' (see section Bioinformatics)

### 4.2 Microsatellite identification and investigation

The microsatellite search was done with the software SciRoKo 3.3 [20]. The following settings were used: mismatched SSR-search with a fixed mismatch penalty; minimum score: 15; fixed mismatch penalty: 5; minimum SSR-seed length: 8; minimum SSR-seed repeats: 3; max mismatches at once: 5; If not denoted otherwise, a *d*_*max *_(maximum distance between adjacent microsatellites as to account as compounded) of 10 bp was used. All microsatellite motifs and SSR-couple motifs were standardized as described by Kofler et al. [20] [see also Additional file 1]. Compound microsatellites and SSR-couples in which all individual microsatellites share the same motif were not considered.

Since the content of the letter 'N' varies between 4 – 20% in the pseudochormosomes of the eight taxa, only the letters 'A','T','C' and 'G' were considered to calculate the sequence length dependent variables (e.g SSR density or compound microsatellite density). The sequence length dependent variables were not adjusted for the cds, 5'-UTR and 3'-UTR.

The microsatellite search results generated with the software SciRoKo were processed with a number of console applications. All console applications were written in C# or Perl. All programs can be obtained from the corresponding author upon request.

### 4.3 Statistics

Calculation of the expected number of SSR heterocouples, i.e. pairs of microsatellites not sharing the same motif, are based on a random distribution of microsatellites within DNA sequence space. The expected number of SSR heterocouples (*C*_*he*.*exp*_: equation 2) was estimated, by calculating the total number of expected SSR-couples (*C*_*exp*_: equation 1) and subtracting, for each microsatellite motif, the expected number of SSR homocouples (equations 1 & 2), i.e pairs of microsatellites sharing the same motif. The overrepresentation (*Or*: equation 3) is calculated by dividing the observed number of microsatellite heterocouples by the expected one:

(1) $$C_{exp}(m)=\frac{d_{max}*m^{2}}{G_{L}-M*\mu_{L}}$$

(2) $$C_{he.exp}=C_{exp}(M)-\sum_{i=1}^{i=p}C_{exp}(m_{i})$$

(3) $$Or=\frac{C_{he.obs}}{C_{he.exp}}$$

The parameters are: *C*_*exp *_expected number of SSR-couples [count]; *G*_*L *_length of the used DNA sequence, not considering the 'N'-letters [bp]; *M *total number of microsatellites [counts]; *μ*_*L *_average length of a microsatellite [bp]; *d*_*max *_maximum distance between adjacent microsatellites as to account as compounded [bp]; *m*, *m*_*i *_number of microsatellites having the specified motif [counts]; *C*_*he*.*exp *_expected number of SSR heterocouples [counts]; *C*_*he*.*obs *_observed number of microsatellite heterocouples [counts]; *p *partially standardized microsatellite motifs [count]; *Or *overrepresentation of microsatellite heterocouples [ratio]. To calculate the overrepresentation for individual microsatellite heterocouples of the form [m1]_*n *_[m2]_*m *_the following equation was used:

(4) $$C_{he.exp}(m1,m2)=\frac{2*d_{max}*m1*m2}{G_{L}-M*\mu_{L}}$$

All parameters are as described above, except for the frequency of the first motif (*m*1) and the frequency of the second motif (*m*2). To test whether the observed number of SSR heterocouples significantly deviates from the expected one, a both sided Poisson Distribution was used and cumulative probabilities were calculated for *P*(*x *≥ *C*_*he*.*obs*_).

To identify the parameters governing compound microsatellite density we determined the SSR and the compound microsatellite density along the chromosomes of *H. sapiens*, *P. troglodytes *and *M. musculus *with a sliding window approach. To avoid statistical bias we used a non-overlapping sliding window approach, setting both the window size and the step size to 5 Mbp. Values representing not-sequenced tracts like ends of chromosomes or centromeres were removed prior to statistical analysis. We categorized the data for each sliding window according the criteria species and chromosome. To test the influence of recombination we categorized the chromosomes in two groups, Y-chromosomes and chromosomes other than Y. The 'Categorial Regression Test' (CatReg) test was done with SPSS 15.0. The influence of recombination was tested separately by using the two groups 'Y' and 'not-Y' instead of the category chromosome. The data used for CatReg test can be found in Additional file 3. To further test the influence of recombination to compound microsatellite denisty we used the recombination map *H. sapiens *as published by Kong et al. [21]. We used a Perl script to determine the microsatellite cluster density and the recombination frequency for each sliding window. Correlation was calculated using Microsoft Excel. The Perl script as well as the resulting raw-data can be found in Additional file 4.

## Authors' contributions

RK, CS and TL designed the study. RK wrote the software and conducted the bioinformatic analysis. EL conducted the statistic analysis. RK, CS, EL and TL wrote the manuscript.

## Supplementary Material

Additional file 1 Standardization of microsatellite motifs and compound microsatellite motifs. Describes in detail, the methods used in this publication, for the standardization of microsatellites and compound microsatellites. Click here for file

Additional file 2 Additional tables. Contains the additional tables S1 – S11. Click here for file

Additional file 3 CatReg raw data. Contains the raw data used for the CatReg-test. Click here for file

Additional file 4 Recombination vs compound microsatellite density. Contains the raw data for calculating the correlation between recombination and compound microsatellite density. Additonally contains the source code of the perl script used for calculating this raw data. Click here for file

Additional file 5 Significant overrepresentation of SSR-couples in all eight species. Contains the expected number of SSR-couples, the observed number of SSR-couples and the significance of the overrepresentation. The analysis has been conducted for each sliding window (size 5 Mbp) in all eight species. Click here for file
