# Supplementary material for: Survey of microsatellite clustering in eight fully sequenced species sheds light on the origin of compound microsatellites
Source: BMC Genomics. 2008 Dec 17;9:612. doi: 10.1186/1471-2164-9-612 (PMC2644718; doi:10.1186/1471-2164-9-612)
Supplement: Additional file 1 — Standardization of microsatellite motifs and compound microsatellite motifs. Describes in detail, the methods used in this publication, for the standardization of microsatellites and compound microsatellites. [file 1471-2164-9-612-S1.pdf]

File S1: Standardization of microsatellite motifs and compound microsatellite motifs
Standardization of microsatellite motifs

Standardization of microsatellite motifs is an important prerequisite for categorizing and comparing of microsatellites. Two standardization intensities were used: partial and full standardization. In the partial standardization, only variations of the motif occuring in a one DNA strand are considered, whereas for the full standardization also the motifs from the reverse complement strand are considered. For example, a poly-CTT , a poly-TCT and a poly-TTC tract have the same partially standardised motif: TTC. By contrast the reverse complement tracts of those simple sequences poly-AAG, poly-AGA and poly-GAA have the partially standardized motif AAG. It is obvious that the poly-AAG and poly-TTC tracts merely represent the plus and minus strand of the same microsatellite, thus, upon full standardization both microsatellite tracts have the same fully standardized motif: AAG.
The identification of the standardized motif is an arbitrary process and has to follow some simple rules. We introduced a 'ATCG-rule': the standardized motif is the variation of a microsatellite motif, which has, within the constrains of the applied standardization intensity, the most 'A'-nucleotides at the beginning (e.g.: AT instead of TA), subsequently the 'T' (e.g.: ATAC instead of ACAT), the 'C' (e.g.: CG instead of GC) and finally the 'G'-nucleotides are considered. This principle is automatically applied in the software SciRoKo (Kofler *et al.*, 2007).

Standardization of SSR-couple motifs

Standardization of SSR-couples (pairs of adjacent ($d \leq d_{max}$ microsatellite) is far from trivial. Two different microsatellites might be arranged in many different combinations. Consider, for example, a SSR-couple consisting of the two microsatellites $[AC]_n$ and $[AG]_m$. The $[AG]_m$ microsatelltie might be found 5' or 3' of the $[AC]_n$ microsatellite. Furthermore, the poly-AC tract of the $[AC]_n$ microsatellite might either be found on the same DNA strand as the poly-AG tract of the $[AG]_m$ microsatellite, or on the reverse complement strand which contains the poly-TC tract of the $[AG]_m$ microsatellite.
The standardization of SSR-couple motif requires, in addition to the 'ATCG-rule', the introduction of a new rule, the short motifs first rule. If the 5'-3' arrangement is not considered (depending on the applied standardization intensity, see below) the shortest motif is displayed first (e.g.: C-AG). Four standardization intensities can be used to standardize the motifs of SSR-couples. We depict the motifs of SSR-couples as the motif of the first microsatellite separated from the motif of the second microsatellite by the '-' symbol. Additionally, a shortcut for the applied standardization intensity may be added in front of each SSR-couple

motif. This is necessary because the SSR-couple motifs alone do not relate which standardization intensity was used. The following Table shows the four different standardization intensities and a simple example for each category.

- 'F:' found SSR-couple, no standardization is applied. For example 5'-$[GT]_7$-NNN-$[GA]_8$-3' is shown as F:GT-GA
- 'PSS:' partial standardization single strand. Both individual microsatellite motifs are partially standardized, the 5'-3' arrangement is considered. For example 5'-$[GT]_7$-NNN-$[GA]_8$-3' is shown as PSS:TG-AG
- 'PSB:' partial standardization both strands. In addition to the 'PSS' the reverse complement strand 'SSR-Couple' is also considered. Additionally, the 5'-3' arrangement is considered. For example 5'-$[GT]_7$-NNN-$[GA]_8$-3' is shown as PSB:TC-AC
- 'CS:' conformation standardization. The 5'-3' arrangement is not considered anymore. Both individual microsatellite motifs are partially standardized. For example 5'-$[GT]_7$-NNN-$[GA]_8$-3' is depicted as CS:AC-TC
- 'FS:' ful standardization. Both individual microsatellite motifs are fully standardized. For example 5'-$[GT]_7$-NNN-$[GA]_8$-3' is depicted as FS:AC-AG. All SSR-couple motifs shown in this work are fully standardized.

These standardization intensities are automatically applied in the software SciRoKo Kofler *et al.* (2007). The conformation standardized SSR-couple motifs, having both fully standardized microsatellite motifs on the same DNA-strand are said to be in 'plus'-conformation whereas those SSR-couples having the fully standardized motifs on opposite DNA strands are said to be in 'minus'-conformation. For SSR-Couples containing a microsatellite with a self complementary motif only one conformation can be distinguished. Figure 1 demonstrates the relationship of the four different standardization intensities.

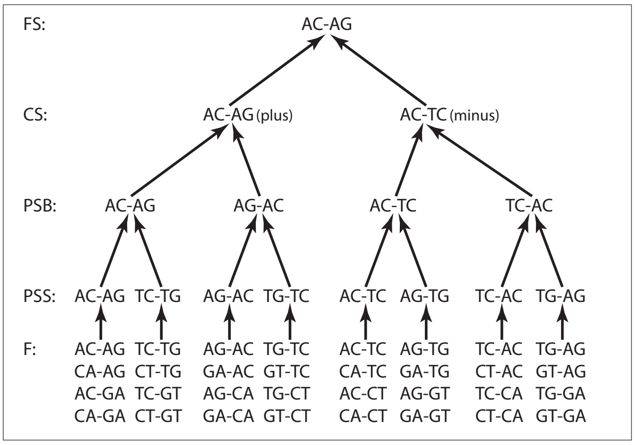

Fig. S 1: Standardization intensities of SSR-couples (automatically applied by SciRoKo). For example, the motif of the SSR-couple 5'-$[CT]_7$-$[GT]_9$-3' is shown as F:CT-GT; F: found motif; PSS: partial standardized single strand; PSB: partially standardized both strands; CS: conformation standardized; FS: fully standardized
