# Supplementary material for: Survey of microsatellite clustering in eight fully sequenced species sheds light on the origin of compound microsatellites
Source: BMC Genomics. 2008 Dec 17;9:612. doi: 10.1186/1471-2164-9-612 (PMC2644718; doi:10.1186/1471-2164-9-612)
Supplement: Additional file 2 — Additional tables. Contains the additional tables S1 – S11. [file 1471-2164-9-612-S2.pdf]

Table S 1: Frequency of compound microsatellites in the 5' and 3' untranslated regions (UTR). SSR: number of microsatellites [count]; comp.: number of compound microsatellites [count]; cSSR: number of individual microsatellites being part of a compound microsatellite [count]; c.p.: percentage of individual microsatellites being part of a compound microsatellite [%]; ms.d.: microsatellite density [SSR/Mbp]; co.d.: compound microsatellite density [comp./Mbp]; *H. sap.: Homo sapiens*; *M. mul.: Macaca mulatta*; *M. mus.: Mus musculus*; *R. nor.: Rattus norvegicus*; *O. anat.: Ornithorhynchus anatinus*; *G. gal.: Gallus gallus*; *D. rerio.: Danio rerio*; *D. mel.: Drosophila melanogaster*

	5'-UTR						3'-UTR					
species	SSR	comp.	cSSR	c.p.	ms.d.	co.d.	SSR	comp.	cSSR	c.p.	ms.d.	co.d
H. sap.	2718	78	158	5.8	293.5	8.4	7513	176	371	4.9	240.5	5.6
M. mul.	860	17	34	4.0	226.8	4.5	2235	34	68	3.0	192.2	2.9
M. mus.	2261	76	163	7.2	330.5	11.1	10396	561	1205	11.6	372.0	20.1
R. nor.	506	8	16	3.2	153.2	2.4	1815	78	160	8.8	258.6	11.1
O. anat.	40	1	2	5.0	252.4	6.3	129	1	2	1.6	172.0	1.3
G. gal.	387	11	23	5.9	325.3	9.3	964	16	33	3.4	171.4	2.9
D. rerio	235	4	8	3.4	195.1	3.3	2023	38	78	3.9	412.8	7.8
D. mel.	1529	39	78	5.1	403.4	10.3	2152	43	91	4.2	465.3	9.3

Table S 2: Influence of the microsatelltite search settings on the frequency of compound microsatellites in the whole genome and in the coding sequence (cds). search: used microsatellite search setting; S15: search for imperfect microsatellites, required score 15; S20: search for imperfect microsatellites, required score 20; S30: search for imperfect microsatellites, required score 30; L15: search for perfect microsatellites, required microsatellite length 15; SSR: number of microsatellites [count]; comp.: number of compound microsatellites [count]; cSSR: number of individual microsatellites being part of a compound microsatellite [count]; c.p.: percentage of individual microsatellites being part of a compound microsatellite [%]; ms.d.: microsatellite density [SSR/Mbp]; co.d.: compound microsatellite density [comp./Mbp];

		whole genome						cds					
species	search	SSR	comp.	cSSR	c.p.	ms.d.	co.d.	SSR	comp.	cSSR	c.p.	ms.d.	co.d
H. sap.	S15	1169530	59792	129848	11.1	413.0	21.1	4965	104	233	4.7	77.4	1.6
	S20	533781	19835	42359	7.9	188.5	7.0	1852	30	60	3.2	28.9	0.5
	S30	148233	4520	9489	6.4	52.3	1.6	364	5	10	2.8	5.7	0.1
	L15	1140608	53693	119192	10.5	402.7	19.0	4452	80	180	4.0	69.4	1.3
M. mul.	S15	1178381	61407	134455	11.4	445.3	23.2	3638	64	139	3.8	71.3	1.3
	S20	560975	21523	47007	8.4	212.0	8.1	1421	25	55	3.9	27.9	0.5
	S30	145843	5740	12383	8.5	55.1	2.2	240	10	21	8.8	4.7	0.2
	L15	1156605	56509	127678	11.0	437.1	21.4	3331	51	118	3.5	65.3	1.0
M. mus.	S15	1574180	173535	398361	25.3	617.9	68.1	3995	95	202	5.1	72.5	1.7
	S20	1004216	106505	240813	24.0	394.2	41.8	1634	34	74	4.5	29.7	0.6
	S30	599913	55686	121539	20.3	235.5	21.9	407	9	19	4.7	7.4	0.2
	L15	1651039	165166	399941	24.2	648.1	64.8	3601	79	174	4.8	65.4	1.4
R. nor.	S15	1307474	133120	291304	22.3	527.8	53.7	1883	92	226	12.0	92.6	4.5
	S20	854201	81448	173568	20.3	344.9	32.9	977	62	145	14.8	48.0	3.1
	S30	549654	38430	79186	14.4	221.9	15.5	474	29	64	13.5	23.3	1.4
	L15	1349861	122418	281760	20.9	545.0	49.4	1895	88	233	12.3	93.2	4.3
O. anat.	S15	133984	1913	3969	3.0	327.2	4.7	1535	16	34	2.2	42.8	0.5
	S20	37913	555	1162	3.1	92.6	1.4	288	4	8	2.8	8.0	0.1
	S30	7352	115	240	3.3	18.0	0.3	69	1	2	2.9	1.9	0.0
	L15	123136	1558	3288	2.7	300.7	3.8	1469	16	32	2.2	41.0	0.5
G. gal.	S15	233896	8532	17989	7.7	237.5	8.7	1889	36	77	4.1	58.3	1.1
	S20	84703	2614	5673	6.7	86.0	2.7	532	10	22	4.1	16.4	0.3
	S30	17946	1033	2279	12.7	18.2	1.1	74	3	6	8.1	2.3	0.1
	L15	225984	8045	17629	7.8	229.5	8.2	1658	21	45	2.7	51.2	0.7
D. rerio	S15	1048258	94159	225069	21.5	688.1	61.8	3215	86	180	5.6	72.0	1.9
	S20	598925	57277	133966	22.4	393.2	37.6	1205	25	52	4.3	27.0	0.6
	S30	349068	28067	63452	18.2	229.2	18.4	317	2	5	1.6	7.1	0.0
	L15	1107628	87463	220899	19.9	727.1	57.4	2907	67	140	4.8	65.1	1.5
D. mel.	S15	44600	714	1457	3.3	376.9	6.0	4168	105	213	5.1	145.6	3.7
	S20	15471	113	230	1.5	130.7	1.0	1591	14	29	1.8	55.6	0.5
	S30	1724	4	8	0.5	14.6	0.0	212	0	0	0.0	7.4	0.0
	L15	41264	645	1317	3.2	348.7	5.5	3573	81	165	4.6	124.8	2.8

Table S 3: Influence of d_{max}, for the whole genome of the eight species. d_{max}: maximum distance between two adjacent microsatellites in order to still account as clustered [bp]; comp.: number of compound microsatellites [count]; cSSR: number of microsatellites being part of a compound microsatellite [count]; c.p.: percentage of microsatellites being part of a compound microsatellite with respect to all microsatellites [%];

	d_{max} [bp]	1	2	3	4	5	6	7	8	9	10	20	30	40	50	100	200	400
	comp.	40159	45085	48120	50660	52849	54977	56332	57605	58663	59792	66454	70233	73173	75750	85767	116657	160716
H. sap.	cSSR	84171	94960	101773	107552	112722	117701	121002	124178	126916	129848	148756	160388	169459	177199	205269	282155	403161
	c.p.	7.2	8.1	8.7	9.2	9.6	10.1	10.4	10.6	10.9	11.1	12.7	13.7	14.5	15.2	17.6	24.1	34.5
	comp.	42581	47281	50108	52612	54686	56719	58064	59333	60346	61407	67845	71438	74245	76738	86783	118826	164449
M. mul.	cSSR	90250	100680	107086	112870	117744	122613	125948	129083	131735	134455	152220	162814	170798	177513	202810	279667	402673
	c.p.	7.7	8.5	9.1	9.6	10.0	10.4	10.7	11.0	11.2	11.4	12.9	13.8	14.5	15.1	17.2	23.7	34.2
	comp.	125826	136390	143639	150823	157572	162183	165342	168396	170911	173535	189170	197858	204080	209306	231413	269865	317913
M. mus.	cSSR	277327	303158	320967	338664	355274	367413	375856	384114	391020	398361	444964	471945	491404	507500	572444	689234	863673
	c.p.	17.6	19.3	20.4	21.5	22.6	23.3	23.9	24.4	24.8	25.3	28.3	30.0	31.2	32.2	36.4	43.8	54.9
	comp.	94045	103361	109345	115098	119908	123948	126374	128886	130929	133120	146147	153485	158731	162853	179931	209164	248240
R. nor.	cSSR	199005	220211	233829	247302	258401	268200	274189	280489	285608	291304	327680	349729	365850	378664	432417	519493	652846
	c.p.	15.2	16.8	17.9	18.9	19.8	20.5	21.0	21.5	21.8	22.3	25.1	26.8	28.0	29.0	33.1	39.7	49.9
	comp.	1210	1363	1479	1578	1658	1714	1767	1817	1868	1913	2322	2588	2862	3118	4536	8082	13651
O. anat.	cSSR	2477	2800	3041	3247	3418	3541	3657	3763	3871	3969	4876	5498	6129	6706	9958	17911	31268
	c.p.	1.9	2.1	2.3	2.4	2.6	2.6	2.7	2.8	2.9	3.0	3.6	4.1	4.6	5.0	7.4	13.4	23.3
	comp.	5284	6067	6526	6927	7298	7631	7888	8154	8338	8532	9903	10818	11594	12264	15033	19545	26864
G. gal.	cSSR	10984	12628	13621	14482	15272	16001	16559	17139	17560	17989	21111	23210	24977	26516	32789	43294	61726
	c.p.	4.7	5.4	5.8	6.2	6.5	6.8	7.1	7.3	7.5	7.7	9.0	9.9	10.7	11.3	14.0	18.5	26.4
	comp.	72758	78284	81514	84734	86920	89002	90533	91927	93060	94159	100928	104882	108506	111767	126116	150801	173704
D. rerio	cSSR	162084	176792	185425	194872	201193	207682	212320	217070	220897	225069	256809	278833	297190	313962	383001	491447	610786
	c.p.	15.5	16.9	17.7	18.6	19.2	19.8	20.3	20.7	21.1	21.5	24.5	26.6	28.4	30.0	36.5	46.9	58.3
	comp.	291	394	437	482	524	565	609	642	680	714	911	1082	1267	1496	2553	4561	6788
D. mel.	cSSR	588	796	883	974	1060	1144	1238	1307	1387	1457	1870	2227	2613	3087	5410	10257	16459
	c.p.	1.3	1.8	2.0	2.2	2.4	2.6	2.8	2.9	3.1	3.3	4.2	5.0	5.9	6.9	12.1	23.0	36.9

Table S 4: Influence of d_{max}, for the coding sequence of the eight species. d_{max}: maximum distance between two adjacent microsatellites in order to still account as clustered [bp]; comp.: number of compound microsatellites [count]; cSSR: number of microsatellites being part of a compound microsatellite [count]; c.p.: percentage of microsatellites being part of a compound microsatellite with respect to all microsatellites [%];

	d_{max} [bp]	1	2	3	4	5	6	7	8	9	10	20	30	40	50	100	200	400
	comp.	61	72	76	81	82	87	89	96	98	104	137	156	162	178	230	279	339
H. sap.	cSSR	131	159	168	179	183	197	201	217	221	233	313	363	376	411	552	683	843
	c.p.	2.6	3.2	3.4	3.6	3.7	4.0	4.1	4.4	4.5	4.7	6.3	7.3	7.6	8.3	11.1	13.8	17.0
	comp.	44	46	50	52	53	53	55	59	61	64	79	95	96	104	142	164	207
M. mul.	cSSR	95	99	108	112	115	116	120	128	133	139	175	213	216	233	333	394	498
	c.p.	2.6	2.7	3.0	3.1	3.2	3.2	3.3	3.5	3.7	3.8	4.8	5.9	5.9	6.4	9.2	10.8	13.7
	comp.	56	62	66	73	78	79	80	90	93	95	124	148	166	174	224	271	327
M. mus.	cSSR	121	133	141	155	165	168	171	191	197	202	271	322	366	388	501	621	758
	c.p.	3.0	3.3	3.5	3.9	4.1	4.2	4.3	4.8	4.9	5.1	6.8	8.1	9.2	9.7	12.5	15.5	19.0
	comp.	61	70	74	78	80	86	85	92	92	92	110	120	125	128	147	155	176
R. nor.	cSSR	143	165	176	184	188	203	204	219	221	226	284	317	339	354	427	468	538
	c.p.	7.6	8.8	9.4	9.8	10.0	10.8	10.8	11.6	11.7	12.0	15.1	16.8	18.0	18.8	22.7	24.9	28.6
	comp.	11	12	12	12	12	12	14	16	16	16	23	25	26	27	35	48	68
O. anat.	cSSR	23	25	25	25	25	25	30	34	34	34	50	56	60	62	83	122	173
	c.p.	1.5	1.6	1.6	1.6	1.6	1.6	2.0	2.2	2.2	2.2	3.3	3.7	3.9	4.0	5.4	8.0	11.3
	comp.	18	25	27	28	31	32	32	33	34	36	42	47	50	54	72	84	100
G. gal.	cSSR	41	55	59	61	67	69	69	71	73	77	94	105	113	123	172	202	241
	c.p.	2.2	2.9	3.1	3.2	3.6	3.7	3.7	3.8	3.9	4.1	5.0	5.6	6.0	6.5	9.1	10.7	12.8
	comp.	49	57	61	62	64	68	68	70	82	86	93	112	128	136	169	220	279
D. rerio	cSSR	103	119	127	129	133	141	143	147	172	180	207	245	286	306	402	538	691
	c.p.	3.2	3.7	4.0	4.0	4.1	4.4	4.5	4.6	5.4	5.6	6.4	7.6	8.9	9.5	12.5	16.7	21.5
	comp.	60	70	78	81	85	89	94	97	100	105	123	136	155	166	237	338	440
D. mel.	cSSR	123	143	159	165	173	181	191	197	203	213	255	285	325	350	537	868	1218
	c.p.	3.0	3.4	3.8	4.0	4.2	4.3	4.6	4.7	4.9	5.1	6.1	6.8	7.8	8.4	12.9	20.8	29.2

Table S 5: Influence of d_{max}, for the 5' untranslated region of the eight species. d_{max}: maximum distance between two adjacent microsatellites in order to still account as clustered [bp]; comp.: number of compound microsatellites [count]; cSSR: number of microsatellites being part of a compound microsatellite [count]; c.p.: percentage of microsatellites being part of a compound microsatellite with respect to all microsatellites [%];

	d_{max} [bp]	1	2	3	4	5	6	7	8	9	10	20	30	40	50	100	200	400
	comp.	49	58	61	62	69	69	70	75	78	78	99	115	132	139	185	244	276
H. sap.	cSSR	98	116	122	126	140	140	142	152	158	158	205	240	276	295	394	533	611
	c.p.	3.6	4.3	4.5	4.6	5.2	5.2	5.2	5.6	5.8	5.8	7.5	8.8	10.2	10.9	14.5	19.6	22.5
	comp.	7	8	10	12	13	16	16	16	16	17	25	28	31	35	42	53	58
M. mul.	cSSR	14	16	20	24	26	32	32	32	32	34	50	56	62	70	84	108	120
	c.p.	1.6	1.9	2.3	2.8	3.0	3.7	3.7	3.7	3.7	4.0	5.8	6.5	7.2	8.1	9.8	12.6	14.0
	comp.	40	53	57	59	59	64	65	70	76	76	100	108	116	127	161	205	229
M. mus.	cSSR	85	111	123	127	127	137	139	150	162	163	216	235	253	276	355	452	519
	c.p.	3.8	4.9	5.4	5.6	5.6	6.1	6.2	6.6	7.2	7.2	9.6	10.4	11.2	12.2	15.7	20.0	23.0
	comp.	5	7	7	7	8	8	8	8	8	8	9	12	15	17	22	29	34
R. nor.	cSSR	10	14	14	14	16	16	16	16	16	16	20	27	33	37	47	64	74
	c.p.	2.0	2.8	2.8	2.8	3.2	3.2	3.2	3.2	3.2	3.2	4.0	5.3	6.5	7.3	9.3	12.7	14.6
	comp.	1	1	1	1	1	1	1	1	1	1	1	1	1	1	1	1	1
O. anat.	cSSR	2	2	2	2	2	2	2	2	2	2	2	2	2	2	2	2	2
	c.p.	5.0	5.0	5.0	5.0	5.0	5.0	5.0	5.0	5.0	5.0	5.0	5.0	5.0	5.0	5.0	5.0	5.0
	comp.	4	7	9	9	9	10	10	11	11	11	18	19	21	22	30	35	36
G. gal.	cSSR	8	14	18	18	18	20	20	22	23	23	37	40	44	46	62	72	77
	c.p.	2.1	3.6	4.7	4.7	4.7	5.2	5.2	5.7	5.9	5.9	9.6	10.3	11.4	11.9	16.0	18.6	19.9
	comp.	2	2	2	3	3	3	3	4	4	4	4	5	5	5	6	11	12
D. rerio	cSSR	4	4	4	6	6	6	6	8	8	8	8	10	10	10	12	22	25
	c.p.	1.7	1.7	1.7	2.6	2.6	2.6	2.6	3.4	3.4	3.4	3.4	4.3	4.3	4.3	5.1	9.4	10.6
	comp.	20	21	24	25	28	28	36	36	38	39	47	52	64	75	102	146	185
D. mel.	cSSR	40	42	48	50	56	56	72	72	76	78	95	105	129	152	213	318	417
	c.p.	2.6	2.8	3.1	3.3	3.7	3.7	4.7	4.7	5.0	5.1	6.2	6.9	8.4	9.9	13.9	20.8	27.3

Table S 6: Influence of d_{max}, for the 3' untranslated region of the eight species. d_{max}: maximum distance between two adjacent microsatellites in order to still account as clustered [bp]; comp.: number of compound microsatellites [count]; cSSR: number of microsatellites being part of a compound microsatellite [count]; c.p.: percentage of microsatellites being part of a compound microsatellite with respect to all microsatellites [%];

	d_{max} [bp]	1	2	3	4	5	6	7	8	9	10	20	30	40	50	100	200	400
	comp.	111	122	140	150	159	163	168	168	173	176	199	216	222	239	311	449	687
H. sap.	cSSR	230	253	290	311	332	341	351	353	364	371	419	457	472	509	670	981	1518
	c.p.	3.1	3.4	3.9	4.1	4.4	4.5	4.7	4.7	4.8	4.9	5.6	6.1	6.3	6.8	8.9	13.1	20.2
	comp.	16	22	23	24	27	29	30	31	34	34	44	51	55	56	78	119	149
M. mul.	cSSR	32	44	46	48	54	58	60	62	68	68	88	102	110	112	156	255	324
	c.p.	1.4	2.0	2.1	2.2	2.4	2.6	2.7	2.8	3.0	3.0	3.9	4.6	4.9	5.0	7.0	11.4	14.5
	comp.	384	427	451	474	492	506	526	540	549	561	637	692	741	768	878	1121	1438
M. mus.	cSSR	801	897	950	1005	1048	1082	1124	1157	1177	1205	1394	1519	1629	1697	1964	2542	3371
	c.p.	7.7	8.6	9.1	9.7	10.1	10.4	10.8	11.1	11.3	11.6	13.4	14.6	15.7	16.3	18.9	24.5	32.4
	comp.	55	59	63	66	67	69	71	73	76	78	87	102	111	114	129	155	186
R. nor.	cSSR	113	121	129	135	137	141	146	150	156	160	182	213	232	242	275	338	428
	c.p.	6.2	6.7	7.1	7.4	7.6	7.8	8.0	8.3	8.6	8.8	10.0	11.7	12.8	13.3	15.2	18.6	23.6
	comp.	0	0	0	1	1	1	1	1	1	1	1	2	2	2	2	4	4
O. anat.	cSSR	0	0	0	2	2	2	2	2	2	2	2	4	4	4	4	8	8
	c.p.	0.0	0.0	0.0	1.6	1.6	1.6	1.6	1.6	1.6	1.6	1.6	3.1	3.1	3.1	3.1	6.2	6.2
	comp.	4	5	7	7	9	11	13	16	16	16	19	24	26	26	37	46	62
G. gal.	cSSR	8	10	14	14	18	22	26	32	32	33	39	49	53	53	78	96	137
	c.p.	0.8	1.0	1.5	1.5	1.9	2.3	2.7	3.3	3.3	3.4	4.1	5.1	5.5	5.5	8.1	10.0	14.2
	comp.	23	24	29	30	31	32	34	35	35	38	44	46	52	58	99	144	207
D. rerio	cSSR	47	50	60	62	64	66	70	72	72	78	91	98	111	123	210	314	478
	c.p.	2.3	2.5	3.0	3.1	3.2	3.3	3.5	3.6	3.6	3.9	4.5	4.8	5.5	6.1	10.4	15.5	23.6
	comp.	17	25	27	31	33	34	39	39	42	43	58	70	82	96	149	233	304
D. mel.	cSSR	34	50	54	63	69	71	81	82	88	91	122	146	170	199	328	530	742
	c.p.	1.6	2.3	2.5	2.9	3.2	3.3	3.8	3.8	4.1	4.2	5.7	6.8	7.9	9.3	15.2	24.6	34.5

Table S 7: Size distribution of compound microsatellites in the 5-UTR and 3'-UTR. The size refers to the number of individual microsatellites composing the compound microsatellite. All values are in counts. c.s.: compound microsatellite size [#SSR/compound]

	5'-UTR				3'-UTR			
	2	3	4	>4	2	3	4	>4
H. sap.	76	2	0	0	160	14	1	1
M. mul.	17	0	0	0	34	0	0	0
M. mus.	68	5	3	0	493	57	8	3
R. nor.	8	0	0	0	74	4	0	0
O. anat.	1	0	0	0	1	0	0	0
G. gal.	10	1	0	0	15	1	0	0
D. rerio	4	0	0	0	37	0	1	0
D. mel.	39	0	0	0	38	5	0	0

Table S 8: Overrepresentation of SSR-couples and statistical significance thereof in the 5'-UTR and 3'-UTR. obs.: observed number of SSR-couples [count]; exp.: expected number of SSR-couples with respect to a random distribution of microsatellites within DNA sequence space [count]; or.: overrepresentation of SSR-couples [ratio]; P: statistical significance using a Poisson Distribution

	5'-UTR				3'-UTR			
	obs.	exp.	or.	P	obs.	exp.	or.	P
H. sap.	80	7	11	5E-54	194	16	12	0[1]
M. mul.	17	2	9	1E-12	34	3.93	8	1E-21
M. mus.	86	7	12	9E-62	642	37	17	0[1]
R. nor.	8	1	11	1E-07	82	4	18	4E-73
O. anat.	1	0.1	10	4E-03	1	0.2	5	2E-02
G. gal.	12	1	10	7E-10	17	2	11	8E-14
D. rerio	4	0.4	9	8E-05	40	8	5	6E-17
D. mel.	39	6	7	4E-20	48	9	5	1E-19

[1] $p < 1E - 99$

Table S 9: Features and probable genesis of the most abundant SSR-couples in the 5'-UTR. motif: SSR-couple motif (see text); obs.: observed number of SSR-couples having the given motifs [count]; exp.: expected number of SSR-couples [count]; or.: overrepresentation [ratio]; %plus: percent of the SSR-couples found in the plus-conformation (see Text) [%]. Values in brackets indicate that only the specified conformation is feasible. gen.: suggested genesis of the SSR-couple: c: chance; r: recombination; s: slippage; ?: unknown; -: due to low frequency not annotated

	H. sapiens					*M. mulatta*			
motif	obs.	or.	%plus	gen.	motif	obs.	or.	%plus	gen.
AGC-CCG	14	50	57	s	ACTG-AGCC	2	$> 10^4$	0	-
AC-AG	8	498	13	s	AGC-AGG	2	103	100	-
AGC-AGCCCC	6	1805	100	s/?	AGC-CCG	2	29	50	-
A-AAAG	5	531	100	s	AGG-AAGAGG	2	1747	100	-
AGG-CCG	4	10	50	-	AGG-CCG	2	21	0	-
AAG-AGG	3	398	100	-	A-AG	1	72	100	-
CCG-AGCCTC	2	499	100	-	AAG-AGG	1	437	100	-
AC-CG	2	1847	100	-	CCCCG-CCCGG	1	786	100	-
CCG-CCCGG	2	17	50	-	A-AAAG	1	444	100	-
AGGG-AGGGG	2	653	100	-	ACCGGG-AGGCCG	1	$> 10^4$	100	-
	M. musculus					*R. norvegicus*			
AGC-CCG	8	40	38	s	AC-CG	2	3427	100	-
AC-AG	6	128	33	s	AGGG-CCCCGG	1	3290	0	-
AAG-AGC	6	885	100	s	AGG-CCG	1	30	0	-
AGG-CCG	4	16	50	-	A-AAT	1	7477	0	-
CCG-AGCGGC	4	152	0	-	AC-ACGC	1	3427	100	-
A-AG	3	47	100	-	AGC-ACTGCC	1	3738	0	-
AC-ACAG	3	343	67	-	AGC-AGGC	1	935	100	-
A-AAAG	3	253	100	-	-	-	-	-	-
CCG-CCCG	2	13	100	-	-	-	-	-	-
CCG-ACCGCC	2	103	100	-	-	-	-	-	-
	O. anatinus					*G. gallus*			
ATCG-AGCG	1	7884	100	-	ATCC-ACGG	2	6565	0	-
-	-	-	-	-	CCG-ACCGC	2	353	100	-
-	-	-	-	-	CCG-ACGC	1	882	100	-
-	-	-	-	-	CCCGG-AGGCCC	1	1441	100	-
-	-	-	-	-	AGGGC-CCCCG	1	1641	100	-
-	-	-	-	-	CCCCG-CCGCG	1	615	100	-
-	-	-	-	-	CCG-CCCGG	1	22	100	-
-	-	-	-	-	AGC-AGCCGG	1	3693	100	-
-	-	-	-	-	A-AAAG	1	615	100	-
-	-	-	-	-	AAGG-ACGG	1	1969	100	-
	D. rerio					*D. melanogaster*			
AAC-ATC	1	416	100	-	ACCG-AATCG	6	4336	100	s/?
AAAT-AAAG	1	4283	100	-	AATCT-ATCAG	4	$> 10^4$	0	-
AC-AG	1	22	0	-	AAC-AGC	4	33	100	-
AAC-AAG	1	444	100	-	AGC-AGG	2	93	100	-
-	-	-	-	-	ATACG-ATCCG	2	2349	100	-
-	-	-	-	-	AATCG-ATCCG	2	723	0	-
-	-	-	-	-	AACTCG-AACGTG	2	$> 10^4$	100	-
-	-	-	-	-	AG-ACACG	2	1236	100	-
-	-	-	-	-	AT-AATAT	2	1305	100	-
-	-	-	-	-	ATAC-ATATGC	1	7829	100	-

Table S 10: Features and probable genesis of the most abundant SSR-couples in the 3'-UTR. motif: SSR-couple motif (see text); obs.: observed number of SSR-couples having the given motifs [count]; exp.: expected number of SSR-couples [count]; or.: overrepresentation [ratio]; %plus: percent of the SSR-couples found in the plus-conformation (see Text) [%]. Values in brackets indicate that only the specified conformation is feasible. gen: suggested genesis of the SSR-couple: c: chance; r: recombination; s: slippage (m: multi step slippage); ?: unknown; -: due to low frequency not annotated

	H. sapiens					*M. mulatta*			
motif	obs.	or.	%plus	gen.	motif	obs.	or.	%plus	gen.
AT-AC	36	151	(100)	s	AC-AG	6	179	33	s
AC-AG	25	192	16	s	A-AG	3	29	100	-
A-AAAAG	10	61	100	s	A-AAAAG	3	74	100	-
A-AAAG	9	43	89	s	A-AAAG	3	63	100	-
A-AT	8	13	(100)	s	A-A	2	2	(0)	-
A-AAAAC	6	8	100	s	AAAC-AAAGAG	2	3147	100	-
AC-ACGC	5	493	100	s	A-AAAAC	2	10	50	-
AAGG-AGGG	5	1556	100	s	AT-AC	2	37	(100)	-
AAAG-AAGG	5	971	100	s	AC-ACC	2	287	100	-
A-AAGG	4	37	100	-	AG-AAT	1	201	0	-
	M. musculus					*R. norvegicus*			
AC-AG	136	158	33	s	AC-AG	19	198	21	s
AAAG-AAGG	27	1078	100	s	AC-ACGC	4	486	100	-
A-AAAG	22	62	100	s	AC-CG	4	1092	(100)	-
AG-AGGG	21	325	100	s	A-AAAG	4	80	100	-
AT-AC	15	26	(100)	s	AAGG-AGGG	2	1464	100	-
AC-ACGC	14	324	100	s	A-AAGG	2	64	50	-
AC-ACAG	14	68	100	s	A-AT	2	19	(100)	-
AAAC-AAAAC	14	30	100	s	ATCC-ACGG	2	$> 10^4$	0	-
AC-ATAC	12	92	100	s	A-AAAAG	2	100	100	-
A-AAAAC	11	11	100	s	AC-ACAG	2	104	100	-
	O. anatinus					*G. gallus*			
C-ACGC	1	$> 10^4$	100	-	A-AAAAC	2	24	100	-
-	-	-	-	-	AT-AAAC	2	72	(100)	-
-	-	-	-	-	A-AAC	1	25	100	-
-	-	-	-	-	AAT-AGG	1	490	100	-
-	-	-	-	-	A-AAT	1	17	0	-
-	-	-	-	-	AAT-AAAG	1	414	100	-
-	-	-	-	-	AT-AC	1	75	(100)	-
-	-	-	-	-	AAC-AAAAC	1	111	100	-
-	-	-	-	-	AATAT-AATAT	1	$> 10^4$	(0)	-
-	-	-	-	-	A-AAG	1	175	100	-
	D. rerio					*D. melanogaster*			
AC-ACGC	5	196	100	s	ACC-AGC	6	556	100	s
AT-AC	4	16	(100)	-	AAC-AGC	5	129	100	s
AC-AG	4	19	0	-	AT-AC	4	9	(100)	-
A-AT	3	11	(100)	-	AT-ATAC	2	14	(100)	-
AT-AAAT	2	48	(100)	-	AGG-ATCCTC	2	6536	0	-
AC-ACGCCC	2	1017	100	-	AGC-ATCCTC	2	1760	0	-
AAT-AAC	2	69	100	-	ATACCC-ACCCAG	2	$> 10^4$	100	-
AAT-AAT	2	19	(0)	-	ATATAC-ATATAG	2	8473	100	-
AT-AG	2	35	(100)	-	AC-AGG	1	29	100	-
A-AG	1	5	100	-	ACAG-ATCGC	1	7625	100	-

Table S 11: Percentage of tri-SSR-compounds having the pattern $[m1]_n[m2]_m[m1]_o$ where m1 and m2 denote individual microsatellite motifs (partially standardized: file S1). For example: $[AC]_9[AG]_7[AC]_{16}$

	tested	hits	%
H. sapiens	5 835	1 964	33.7
M. mulatta	6 382	1 925	29.3
M. musculus	26 012	9 108	35.0
R. norvegicus	15 284	4 856	30.4
O. anatinus	102	44	43.1
G. gallus	594	184	31.0
D. rerio	15 284	7 041	46.1
D. melanogaster	29	9	31.0
